# Molecular validation of anthropophilic Phlebotominae sandflies (Diptera: Psychodidae) in Central Panama

**DOI:** 10.1590/0074-02760190034

**Published:** 2019-08-15

**Authors:** Larissa Dutari, Jose R Loaiza

**Affiliations:** 1 Instituto de Investigaciones Científicas y Servicios de Alta Tecnología, Ciudad del Saber, República de Panamá; 2 Acharya Nagarjuna University, Department of Biotechnology, Guntur, India; 3 Smithsonian Tropical Research Institute, Panama City, Republic of Panama; 4 Universidad de Panamá, Programa Centroamericano de Maestría en Entomología, Ciudad del Panamá, República de Panamá

**Keywords:** species validation, molecular barcodes, *Psychodopygus thula* species complex, *Leishmania* infection, Panama

## Abstract

Six Phlebotominae sand fly species are incriminated as biological vectors of human pathogens in Panama, but molecular corroboration is still needed. We aim at confirming the identity of Phlebotominae species documented as anthropophilic in Panama. Adult sandflies were collected from August 2010 to February 2012 in Central Panama using CDC light traps. Species confirmation was accomplished through molecular barcodes and allied sequences from GenBank. A total of 53,366 sand fly specimens representing 18 species were collected. Five species were validated molecularly as single phylogenetic clusters, but *Psychodopygus thula* depicted two genetically divergent lineages, which may be indicative of cryptic speciation.

In Panama, six anthropophilic (i.e., man-biters) species of Phlebotominae sandflies (Order Diptera, Family Psychodidae) have been implicated as vectors of *Leishmania* parasites, the causing agent of American cutaneous leishmaniasis (ACL).^1^ ^,^ ^2^ ^,^ ^3^ ^,^ ^4^ ^,^ ^5^ ^,^ ^6^ ^,^ ^7^ Some of these taxa are also suspected vectors of *Phlebovirus* pathogens to a broad range of animal hosts, including humans.^8^ ^,^ ^9^ ^,^ ^10^ ^,^ ^11^ *Lutzomyia gomezi*, *Lutzomyia sanguinaria*, *Nyssomyia ylephiletor*, *Nyssomyia trapidoi*, *Psychodopygus panamensis*, and *Psychodopygus thula* are widespread across forested areas of Panama feeding on various animal species depending on habitat quality and host availability.^12^ Earlier taxonomic work using molecular markers supported the specific status of all these taxa in Central Panama, except for *Lu. gomezi*, for which significant lineage divergence was suggested.^6^ Authors hypothesized *Lu. gomezi* to be a cryptic species complex based on the results of phylogenetic analysis using partial DNA sequences of the mitochondrial Cytochrome C Oxidase Subunit One gene (*CO1*).^6^ This finding was of great epidemiological significance because morphologically identified *Lu. gomezi* was found infected with *Leishmania naiffi*, in pristine site at a high infection rate. *L. naiffi* causes cutaneous leishmaniasis (CL) in South America, but had never been reported from the country of Panama before.^6^ A subsequent molecular study, using samples of *Lu. gomezi* from across the entire country, found low levels of genetic differentiation among populations, thus rejecting the hypothesis of linages diversification in this taxon.^13^ Although there seems to be an acceptable level of agreement between morphology and DNA barcoding-based taxonomy for Phlebotominae sand fly species in Panama,^6^ there is still a need to validate species boundaries using samples from additional sites, particularly from Central Panama. Herein, we revisit the molecular identity of Phlebotominae sand fly species documented as man-biters^12^ in Central Panama, including more locations than the ones used in earlier work.^6^ In so doing, we surveyed for sand fly specimens repeatedly in three ecologically distinct areas to account for spatial changes in the community metrics and to look for *Leishmania* parasite infection in the most prevalent sand fly species.

The study was conducted in the lowland tropical rainforest ecosystem of Central Panama, a region formerly known as the Panama Canal Zone. Detailed information on the sampling area, trapping design and effort was published elsewhere.^14^ ^,^ ^15^ Captured sandflies were separated from other insects under a stereoscope, labeled with a unique code, and initially identified using female morphological characters^16^ ^,^ ^17^ and the taxonomic nomenclature by Galati.^18^

Well-preserved specimens were subjected to molecular analysis using the Barcoding region (5’ prime region of the *CO1* gene) (http://www.barcodeoflife.org/). A total of 184 samples from 13 species, initially identified based on morphological characters, were randomly taken from the total collected and processed molecularly to validate their taxonomic identity. DNA extraction, polymerase chain reaction (PCR)-amplification and sequencing were done following standard protocols.^19^ To determine whether our specimens were mistakenly classified or confused with other species within Phlebotominae, we employ Basic Local Alignments Search Tool (http://blast.ncbi.nlm.nih.gov/) to the allied nucleotide *CO1* sequences of Phlebotominae in GenBank, including those reported in Azpurua and collaborators.^6^ We built a Neighbor-Joining (NJ) phylogenetic tree using all these *CO1* sequences in MEGA v.5.1,^20^ with Kimura 2 parameter (K_2_P) distances, and bootstrapped the topology with 1000 replicates to obtain branch support.

In addition, female sandflies were pooled in groups of up to 50 individuals, according to species, trap location, and height, and tested for infection with *Leishmania* parasites. The DNA of pools was extracted using the Biosprint® 96 DNA Blood kit (Qiagen) DNA Blood Kit on a BioSprint® 96 extraction robotic platform (Qiagen). Pooled DNA was then used to amplify the minicircle kinetoplast DNA of *Leishmania* parasite using the PCR, primers, and cycling conditions reported in Montalvo and collaborators.^21^ A second confirmatory PCR, targeting the entire length of the *Leishmania* Hsp70 gene (1,286 base pairs) was conducted following the protocol, primers, and PCR cycling conditions reported in Cardoso da Graça and collaborators.^21^ DNA amplicons representatives of positive samples were subjected to Restriction Fragment Length Polymorphism (RFLP) technique^22^ and identified by comparing of RFLP banding patterns with those published in Montalvo and collaborators.^23^ *Leishmania* infection rate in sandflies was calculated overall and per species using maximum likelihood estimates (MLE) of pooled samples using an online calculation tool available at http:// www.cdc.gov/westnile/resourcepages/mosqSurvSoft.html.

We generated valid DNA barcode sequences for 160 female Phlebotominae samples out of a total of 184 attempted (> 85% success rate). Failures were due to double peaks in the electropherograms recovered in multiple amplification cycles. We removed these samples from further analyses. 102 unique *CO1* sequences or haplotypes were obtained from samples initially assigned to Phlebotominae based on morphology. All these haplotypes were unambiguously aligned and no insertions or deletions were found. The absence of pseudogenes was established by the lack of stop codons, low pairwise divergence and clear electropherograms. Individual length for these *CO1* haplotypes ranged from 615 to 649 base pairs (bp), with a final alignment length of 610 bp (GenBank accession numbers MN257585-MN257605). Phlebotominae *CO1* sequences formed 14 DNA barcode clusters in the NJ phylogenetic tree, of which some matched with 99% homology the barcode dataset of sand fly species previously reported from Barro Colorado Island (BCI) by Azpurua and collaborators.^6^ These clusters were well-supported statistically, with the majority of haplotypes being found in the three sampling areas (Figure). All man-biter Phlebotominae sand fly species, named currently as *Psychodopygus panamensis* (GenBank accession GU001750.1), *Nyssomyia trapidoi* (GU001764.1), *Nyssomyia ylephiletor*, *Lutzomyia gomezi* (GU001739.1) and *Lutzomyia sanguinaria* (GU001757.1) as well as other rare zoophilic taxa *Bichromomyia olmeca bicolor* (GU001743.1), *Trichopygomyia triramula* (GU001767.1), *Pintomyia ovallesi* (GU001746.1), *Pressatia dysponeta* (GU001732.1)*, Psathyromyia aclydifera* (GU001724.1), and *Micropygomyia trinidadensis* (GU001765.1) matched with their corresponding barcode sequence in Azpurua and collaborators,^6^ and therefore, they were validated as single evolutionary units or molecular species through phylogenetic analysis. However, *Ps. thula* (formerly known as *Lutzomyia carrerai thula*) comprised two moderately divergent molecular lineages, which had not been reported earlier. One of these lineages matched with 99% homology the barcode sequence of *Ps. thula* (GenBank accession GU001753.1) described by Azpurua and collaborators,^6^ therefore we call this taxon *Ps. thula sensu stricto*. Another unidentified lineage in the same cluster showed more than 2% genetic distance from *Ps. thula* s.s., hence it may be a different taxonomic unit under incipient speciation (Figure).

**Figure:**
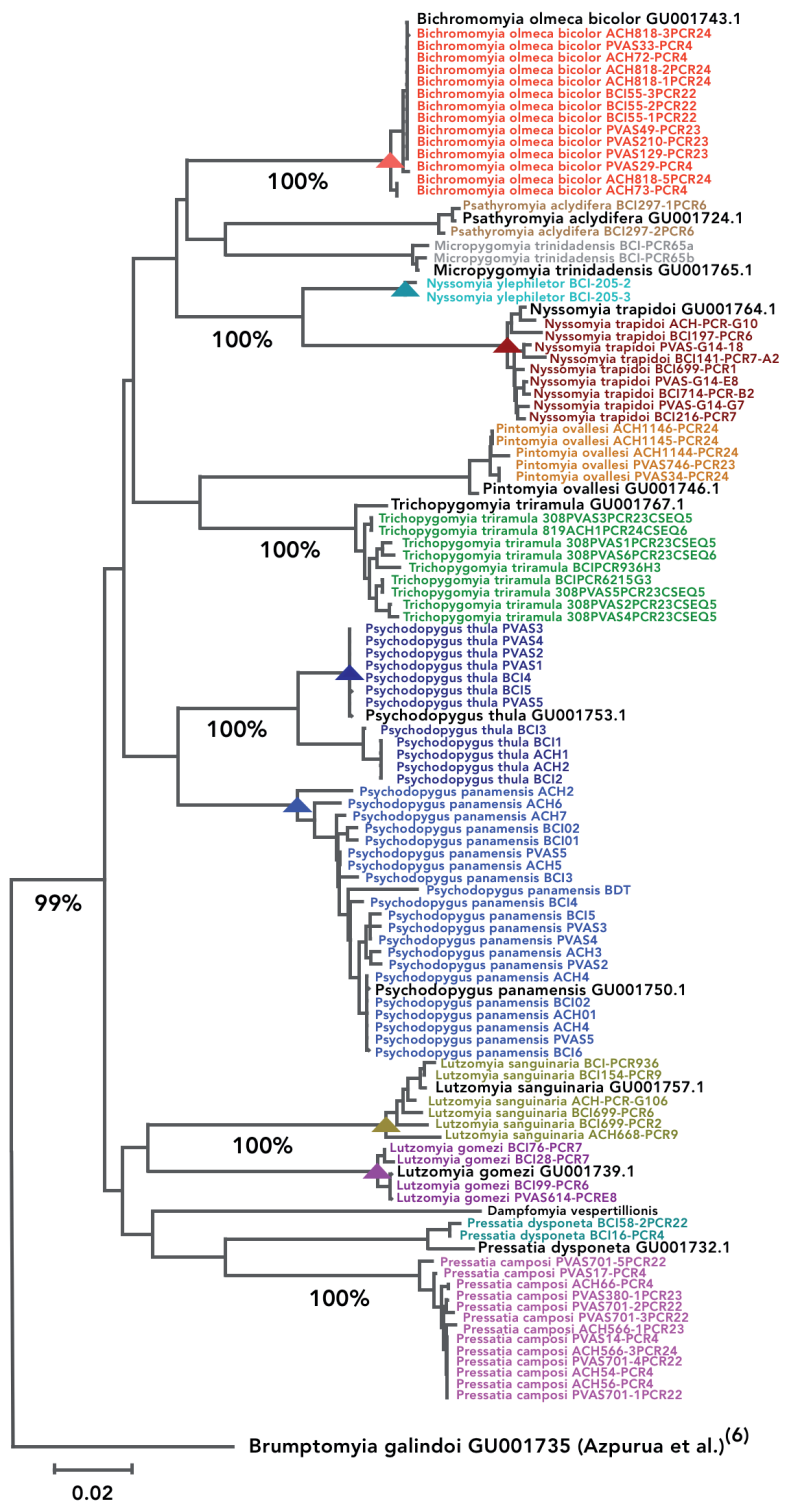
Neighbor-Joining phylogenetic tree using mitochondrial *CO1* gene haplotypes of samples identified morphologically as Phlebotominae sand flies. *Psychodopygus panamensis* (GenBank accession GU001750.1), *Nyssomyia trapidoi* (GU001764.1), *Lutzomyia gomezi* (GU001739.1) *Nyssomyia ylephiletor*, *Lutzomyia sanguinaria* (GU001757.1), *Bichromomyia olmeca bicolor* (GU001743.1), *Trichopygomyia triramula* (GU001767.1), *Pintomyia ovallesi* (GU001746.1), *Pressatia dysponeta* (GU001732.1), *Psathyromyia aclydifera* (GU001724.1), *Micropygomyia trinidadensis* (GU001765.1) and *Psychodopygus thula* (GenBank accession GU001753.1) correspond to barcode sequences in Azpurura et al.,^(6)^ which were used here to validate species taxonomic designations. *Brumptomyia galindoi* (GU001735.1) was used as outgroup. 100% bootstrap values are shown in highly supported molecular clusters. Sequence codes represent the abbreviation of three sampling areas of central Panama: Barro Colorado Island (BCI), Achiote (ACH) and las Pavas (PVAS), plus the polymerase chain reaction (PCR) cycle code (See also maps of the study area previously published in Eastwood et al.^(14)^ and Loaiza et al.^(15)^).

The NJ phylogenetic tree comprised two main clades where samples from the genera *Lutzomyia* and *Pressatia* grouped together in the most basal clade, and away from the other remaining genera in a second derived clade (Figure). Samples assigned to the following genera: *Lutzomyia* (*Lu. gomezi*, *Lu. sanguinaria*), *Psychodopygus* (*Ps. panamensis*, *Ps. thula*), *Nyssomyia* (*Ny. trapidoi*, *Ny. ylephiletor*), *Pressatia* (*Pressatia camposi*, *Pressatia dysponeta*), *Psathyromyia (Psathyromyia carpenteri*), *Bichromomyia* (*Bichromomyia olmeca bicolor*), *Trichopygomyia* (*Trichopygomyia triramula*), *Micropygomyia* (*Micropygomyia trinidadensis*) and *Pintomyia* (*Pintomyia ovallesi*) all clustered jointly with others from the same genera, and were separated from samples in other classes by roughly 6% to 10% genetic distances (Figure).

A total of 53,366 specimens representing 18 species of Phlebotominae sandflies were gathered from three sampling areas of Central Panama. Comparison of Alfa diversity and richness metrics suggest that Phlebotominae sand flies are more diverse and species rich at the ground level of BCI (i.e., A pristine site) (Table I), while its overall relative abundance does not vary significantly among sampling areas (Kruskall-Wallis p = 0.45) or between vertical strata (Mann-Whitney U = 37; p = 0.71). However, when the latter analysis was performed separately at each sampling site, Phlebotominae sand fly relative abundance differed significantly between vertical strata, being more numerous in the understory of BCI (Mann-Whitney U = 89; p = 0.05), but not in Achiote (ACH) (Mann-Whitney U = 146.5, p = 0.63) or in Las Pavas (PVAS) (Mann-Whitney U = 149.5, p = 0.70). The most prevalent species were *Ps. panamensis* (58.63%), *Ny. trapidoi* (14.78%), and *Lu. gomezi* (14.07%) in that order, followed by *Lu. sanguinaria* (3.34%) plus other 13 rare species. *Ps. panamensis* was equally prevalent in all three sampling areas, but the relative abundance of *Ny. trapidoi* and *Lu. gomezi* differed among sites; both species being more prevalent in ACH and PVAS, which are ecologically disturbed areas (Table II).

265 pools representing roughly 11,404 females of five sand fly species (*Ps. panamensis* 159 pools = 7,316 individuals; *Ny. trapidoi* 54 pools = 2215; *Lu. gomezi* 30 pools = 1135; *Lu. sanguinaria* 14 pools = 477 and *Ny. ylephiletor* eight pools = 261) were tested for infection with *Leishmania* parasites. Of these, only one pool of *Ny. trapidoi*, gathered from the understory of the disturbed sites (i.e., ACH), was positive for the presence of *Leishmania* DNA. The species of *Leishmania* in this positive sample could not be identified though, possibly due to insufficient amount of DNA for successful sanger sequencing. Overall and as for *Ny. trapidoi* alone, the *Leishmania* infection rate given by the MLE was 0.09 (per 1,000 sandflies), based on 11,404 individuals and 95% confidence interval.

**TABLE I:** Species composition, diversity and richness community metrics, and relative abundance of Phlebotominae sandflies in three sampling areas of Central Panama

Species	Nomenclature in Galati^(18)^	BCI (undisturbed)			ACH (disturbed)			PVAS (disturbed)		
		N	%	pi	N	%	pi	N	%	pi
*Lutzomyia panamensis*	*Psychodopygus panamensis*	10138	72.16	0.72	12795	55.52	0.56	8358	51.36	0.51
*Lutzomyia carrerai thula*	*Psychodopygus thula*	621	4.42	0.04	17	0.07	0	568	3.49	0.03
*Lutzomyia gomezi*	*Lutzomyia gomezi*	481	3.42	0.03	1682	7.3	0.07	5348	32.87	0.33
*Lutzomyia trapidoi*	*Nyssomyia trapidoi*	1245	8.86	0.09	5605	24.32	0.24	1035	6.36	0.06
*Lutzomyia olmeca bicolor*	*Bichromomyia olmeca bicolor*	143	1.02	0.01	38	0.16	0	81	0.5	0
*Lutzomyia sanguinaria*	*Lutzomyia sanguinaria*	387	2.75	0.03	1389	6.03	0.06	7	0.04	0
*Lutzomyia ylephiletor*	*Nyssomyia ylephiletor*	138	0.98	0.01	1004	4.36	0.04	22	0.14	0
*Lutzomyia aclydifera*	*Psathyromyia aclydifera*	2	0.01	0	0	0	0	0	0	0
*Lutzomyia camposi*	*Pressatia camposi*	1	0.01	0	30	0.13	0	50	0.31	0
*Lutzomyia carpenteri*	*Psathyromyia carpenteri*	0	0	0	386	1.68	0.02	170	1.04	0.01
*Lutzomyia dysponeta*	*Pressatia dysponeta*	44	0.31	0	27	0.12	0	30	0.18	0
*Lutzomyia galindoi*	*Brumptomyia galindoi*	3	0.02	0	0	0	0	0	0	0
*Lutzomyia nordestina*	*Lutzomyia nordestina*	1	0.01	0	0	0	0	0	0	0
*Lutzomyia ovallesi*	*Pintomyia ovallesi*	9	0.06	0	6	0.03	0	1	0.01	0
*Lutzomyia shannoni*	*Psathyromyia shannoni*	92	0.65	0.01	0	0	0	51	0.31	0
*Lutzomyia trinidadensis*	*Micropygomyia trinidadensis*	162	1.15	0.01	1	0	0	0	0	0
*Lutzomyia triramula*	*Trichopygomyia triramula*	583	4.15	0.04	63	0.27	0	551	3.39	0.03
*Lutzomyia vespertilionis*	*Dampfomyia vespertilionis*	0	0	0	1	0	0	0	0	0
Total		14050	100	1	23044	100	1	16272	100	1

ACH: Achiote; BCI: Barro Colorado Island; N: number of sandflies; %: percentage; pi: relative abundance; PVAS: Las Pavas.

**TABLE II:** Community metrics, taxa richness and total abundance of Phlebotominae sandflies in three sampling areas and two vertical strata of Central Panama

Community metrics	BCI (undisturbed)			ACH (disturbed)			PVAS (disturbed)		
	Total	Understory	Canopy	Total	Understory	Canopy	Total	Understory	Canopy
Taxa	16	12	14	14	13	14	13	12	13
Total abundance	14050	1146	12904	23044	11616	23044	16272	8069	16272
Shannon Wiener (H)	1.14	1.1	1.19	1.29	0.93	1.43	1.25	1.23	1.14
Simpson 1_D	0.47	0.44	0.61	0.62	0.4	0.71	0.62	0.57	0.62
Margaleff’s e^H/S	1.57	1.37	1.56	1.29	1.28	1.28	1.24	1.22	1.33

ACH: Achiote; BCI: Barro Colorado Island; PVAS: Las Pavas.

The natural history of both anthropophilic and zoophilic Phlebotominae sand fly species and their roles as vectors of pathogens to humans are well acknowledged in Panama owing to more than 100 years of scientific research.^12^ Nonetheless, in depth studies about the ecology, behavior and control of this medically important group of insects are still challenging due to high species diversity in the Neotropical region plus limitations to identify fresh samples accurately and rapidly. Taxonomic identification of Phlebotominae sand fly in Panama is largely based on adult morphological characters, but morphological keys are often incomplete,^16^ ^,^ ^17^ ^,^ ^18^ and even senior taxonomists cannot sometimes distinguish among separate species, making it difficult to differentiate between vector and non-vectors. To date, only two studies have tested species boundaries in Phlebotominae sand fly with molecular approaches in Panama.^6^ ^,^ ^13^ Azpurura and collaborators^6^ generated molecular barcodes for 18 species of *Lutzomyia* from the forest understory of BCI, using the same sampling procedure used here. Authors suggested that *Lu. gomezi* and *Dampfomyia vespertilionis* were complexes of isomorphic species, while *Ny. trapidoi*, *Ps. panamensis* and *Ps. thula* were nominated as single molecular clusters. Our results based on more sites across Central Panama, including samples from the forest canopy of disturbed and undisturbed areas, contradict this finding and suggests that *Lu. gomezi* represents a single molecular entity, which also agrees with previous efforts to define the taxonomic status of *Lu. gomezi* across Panama.^13^ In contrast, *Ps. thula* is likely two divergent lineages under incipient speciation not reported in earlier research.^6^ Individuals from the two lineages of *Ps. thula* came from BCI, ACH and PVAS, which reinforces the hypothesis of lineage divergence for this taxon. To date, studies about the biology of *Ps. thula* are still incomplete in Panama. Larvae develop in decaying leaves (e.g., forest leaf-litter) of deeply shaded pristine forest environments, while adults use fallen tree trunks and green plants for oviposition, mating and also as diurnal resting sites.^24^ Females of *Ps. thula* are most active at the ground level during the day, when the risk of ACL transmission to animals and humans likely increases by this species.^12^ ^,^ ^25^ However, the role of *Ps. thula* as a vector of *Leishmania (V) panamensis*, the main parasite causing ACL in Panama, has still not been confirmed,^12^ ^,^ ^24^ ^,^ ^26^ and it was not supported by our results either. *Ny. trapidoi* was the only species infected with *Leishmania* parasite in this study, with just one positive pool gathered from the understory of the disturbed site (ACH). Our results partially agree with findings by Azpurura and collaborators^6^ where *Ny. trapidoi* was also found infected with a *Leishmania* parasite known as *L. naiffi*. However, the overall *Leishmania* infection rate in this study was extremely low in comparison to previous work,^6^ ^,^ ^7^ and we could not corroborate the presence of *L. naiffi* in our positive pool either. Consequently, while our outcomes support the transmission involvement of *Ny. trapidoi* for human pathogens in forest environments of Central Panama, these findings must be validated in future studies.

## References

[1] Johnson P, McConmell E, Hertig M (1963). Natural infection of Leptomoned flagellates in Panamanian
Phlebotomus sandflies. Exp Parasit.

[2] Hertig M, McConnell E (1963). Experimental infection of Panamanian Phlebotomus sandflies with
Leishmania. Exp Parasit.

[3] Christensen HA, Herrer A, Telford SR (1969). Leishmania braziliensis s lat. isolated from Lutzomyia panamensis
in Panama. J Parasitol.

[4] Christensen HA, Herrer A, Fairchild GB (1972). Enzootic cutaneous leishmaniasis in eastern Panama II
Entomological investigations. Ann Trop Med Parasitol.

[5] Christensen HA, Herrer A (1980). Development of a Panamanian strain of Leishmania mexicana in
co-indigenous Lutzomyia sanguinaria and Lutzomyia gomezi (Diptera
Psychodidae). J Med Entomol.

[6] Azpurua J, De la Cruz D.Valderrama A.Windsor D (2010). Lutzomyia sandfly diversity and rates of infection by Wolbachia
and an exotic Leishmania species in Barro Colorado Island,
Panama. PLoS Negl Trop Dis.

[7] Saldaña A, Chaves LF, Rigg CA, Wald C, Smucker JE, Calzada JE (2013). Clinical cutaneous leishmaniasis rates are associated with
household Lutzomyia gomezi, Lutzomyia panamensis and Lutzomyia trapidoi
abundance in Trinidad de Las Minas, western Panama. Am J Trop Med Hyg.

[8] Tesh RB, Boshell J, Young DG, Morales A, Carrasquilla CF, Corredor A (1989). Characterization of five new phleboviruses recently isolated from
sand flies in tropical America. Am J Trop Med Hyg.

[9] Tesh RB, Chaniotis BN, Peralta PH, Johnson KM (1974). Ecology of viruses isolated from Panamanian phlebotominae
sandflies. Am J Trop Med Hyg.

[10] Palacios G, Tesh RB, Travassos da Rosa APA.Savji N.Sze W.Jain K (2011). Characterization of the candiru antigenic complex (Bunyaviridae
Phlebovirus), a highly diverse and reassorting group of viruses affecting
humans in Tropical America. J Virol.

[11] Palacios G, Wiley MR, Travassos da Rosa APA.Guzman H.Quiroz E.Savji N (2015). Characterization of the Punta Toro species complex (genus
Phlebovirus, family Bunyaviridae). J Gen Virol.

[12] Dutari LC, Loaiza JR (2014). American cutaneous leishmaniasis in Panama a historical review of
entomological studies on anthropophilic Lutzomyia sand fly
species. Parasit Vectors.

[13] Valderrama A, Tavares MG, Andrade JD (2014). Phylogeography of Lutzomyia gomezi (Diptera Phlebotominae) on the
Panama Isthmus. Parasit Vectors.

[14] Eastwood G, Loaiza JR, Pongsiri MJ, Sanjur OI, Pecor JE, Auguste AJ (2016). Enzootic arbovirus surveillance in forest habitat and
phylogenetic characterization of novel isolates of Gamboa virus in
Panama. Am J Trop Med Hyg.

[15] Loaiza JR, Dutari LC, Rovira JR, Sanjur OI, Laporta G, Pecor J (2017). Disturbance and mosquito diversity in the lowland tropical
rainforest of central Panama. Sci Rep.

[16] Chaniotis BN (1974). Use of external characters for rapid identification of
Phlebotominae sandflies in vector studies. J Med Entomol.

[17] Young DG, Duncan MA (1994). Guide to the identification and geographic distribution of
Lutzomyia sandflies in Mexico, the West Indies, Central and South America
(Diptera Psychodidae). Mem Amer Ent Inst.

[18] Galati EAB (1995). Phylogenetic systematics of Phlebotominae (Diptera: Psychodidae)
with emphasis on American groups. Bol Dir Malariol Ambient.

[19] Loaiza JR, Scott ME, Bermingham E, Sanjur OI, Rovira JR, Dutari LC (2013). Novel genetic diversity within Anopheles punctimacula s l.:
phylogenetic discrepancy between the Barcode cytochrome c oxidase I (COI)
gene and the rDNA second internal transcribed spacer (ITS2). Acta Trop.

[20] Kumar S, Stecher G, Tamura K (2016). MEGA7 Molecular Evolutionary Genetics Analysis Version 7.0 for
Bigger Datasets. Mol Biol Evol.

[21] Montalvo AM, Fraga J, Monzote L, Montano I, De Doncker S, Dujardin JC (2010). Heat-shock protein 70 PCR-RFLP a universal simple tool for
Leishmania species discrimination in the New and Old World. Parasitology.

[22] da Grac¸a GC.Volpini AC.Romero GAS.de Oliveira Neto MP.Hueb
M.Porrozzi R (2012). Development and validation of PCR-based assays for diagnosis of
American cutaneous leishmaniasis and identification of the parasite
species. Mem Inst Oswaldo Cruz.

[23] Christensen HA, Fairchild GB, Herrer A, Johnson CM, Young DG, De Vasquez AM (1983). The ecology of cutaneous leishmaniasis in the Republic of
Panama. J Med Entomol.

[24] Chaniotis BN, Correa MA, Tesh RB, Johnson KM (1972). Diurnal resting sites of Phlebotominae sand flies in Panamanian
tropical forest. J Med Entomol.

[25] Chaniotis BN, Correa MA (1974). Comparative flying and biting activity of Panamanian Phlebotomine
sandflies in mature forest and adjacent open space. J Med Entomol.

[26] Loaiza JR, Rovira JR, Sanjur OI, Zepeda JA, Pecor J, Foley DH (2019). Forest disturbance and insect disease vector risk in the lowland
tropical rainforest of Central Panama. Trop Med Int Health.

